# Sustainable Conversion of Corncob Biomass Waste into High Performance Carbon Materials for Detection of VOCs at Room Temperature

**DOI:** 10.3390/molecules30010110

**Published:** 2024-12-30

**Authors:** Lindokuhle P. Magagula, Clinton M. Masemola, Tshwafo E. Motaung, Nosipho Moloto, Ella C. Linganiso-Dziike

**Affiliations:** 1 Molecular Sciences Institute, School of Chemistry, University of the Witwatersrand, Braamfontein 2050, South Africa; 2 DSI/NRF Centre of Excellence in Strong Materials, University of the Witwatersrand, Braamfontein 2050, South Africa; 3 Department of Chemistry, Sefako Makgatho Health Science University, Medunsa 0204, South Africa

**Keywords:** corncob residue, VOC detection, activated carbon, ethanol sensing, room temperature gas sensing

## Abstract

The demand for reliable, cost-effective, room temperature gas sensors with high sensitivity, selectivity, and short response times is rising, particularly for environmental monitoring, biomedicine, and agriculture. In this study, corncob waste-derived activated carbon (ACC) was combined with CuO nanoparticles and polyvinyl alcohol (PVA) to fabricate ACC/PVA/CuO composites with CuO loadings of 5, 10, and 15 wt.%. The CuO nanoparticles (average size: 21.79 ± 9.88 nm) were successfully incorporated into the ACC matrix, as confirmed by TEM, XRD, and N_2_ adsorption–desorption analyses. Increasing CuO content reduced the specific surface area due to pore blockage but enhanced the composites’ ethanol sensing performance. The ACC/PVA/CuO (15 wt.%) sensor exhibited the highest response and fastest recovery times (125 s and 130 s, respectively, at 100 ppm ethanol), outperforming other composites and pristine ACC. This improvement was attributed to surface defects and increased active sites promoting vapor adsorption and diffusion. These results demonstrate the potential of ACC/PVA/CuO as an effective ethanol sensor at room temperature.

## 1. Introduction

In recent years, the urgency in monitoring air pollution using eco-friendly high-performance materials has motivated researchers to explore prospective and cost-effective materials. This is because of the high demand for simple, responsive, affordable, and stable sensors suitable for environmental monitoring in different fields, such as safety in mining, air pollution control, and firefighting [1]. A variety of materials, including metal/metal oxide nanoparticles, inorganic semiconductors, carbon nanomaterials, and conjugated polymers, have been explored as potential materials for resistive gas sensing applications [2]. Carbon-based materials have large surface area, low power consumptions and good thermal and chemical stability, which are desirable properties in gas sensing [3]. Among carbon-based materials, activated carbon (ACC) has been considered to be the most promising sensing material because of its inner porous structure, large specific surface area, rich surface chemistry, fast adsorption kinetics, chemical stability, the possibility of molding its structure for specific applications and, most importantly, its low-cost material when compared to other forms of carbon, such as carbon nanotubes (CNTs) [4]. ACC is a carbonaceous material that is predominantly amorphous in nature and has a high porous structure with a high surface area and mechanical strength, which depends largely on the precursor material and activation process [5]. However, due to its low sensitivity and conductivity at room temperature, its application in operable sensors is restricted [6]. Modification of the ACC surface is necessary to improve interaction between the sensing material and target gases in order to achieve high gas adsorption at low gas concentrations. Many efforts have been made in order to improve the sensing performance of ACC by introducing surface functional groups, such as carboxyl, carbonyl, hydroxyl, and phenols, or active substances, such as metal oxides, on the ACC surface [6].

Copper oxide nanoparticles (CuO NPs), a type of p-type semiconductor, have a narrow bandgap of 1.2–1.9 eV and exhibit excellent properties, which have been used in the field of catalysis, gas sensors, waste treatment, batteries, food preservation, high temperature superconductors, solar energy conversion, and dye removal [7]. There have been reports demonstrating that different morphologies of CuO NPs are suitable for the detection of volatile organic compounds (VOCs) [8]. However, CuO NP gas sensors require a high operating temperature to achieve an excellent sensing performance, which could lead to high power consumption and drift problems caused by sintering and the diffusion process [9], limiting their ability to function as room temperature gas sensors. Therefore, new methods should be explored to minimize these challenges.

In this work, the focus is on the use of agricultural waste material (corncob) to produce ACC. The ACC was combined with PVA, which acted as a polymer, to stabilize the CuO NPs on ACC. We report room temperature synthesis of CuO NPs and oil bath preparation of ACC/PVA/CuO composites. We further study the VOC gas sensing properties of the composites using ethanol, acetone, 2-butanol, and methanol. The aim of this work was to investigate the application of ACC derived from corncob in VOC sensing. The role of CuO in the room temperature sensing properties of ACC when exposed to different VOCs was also investigated.

## 2. Results

**Figure 1:**
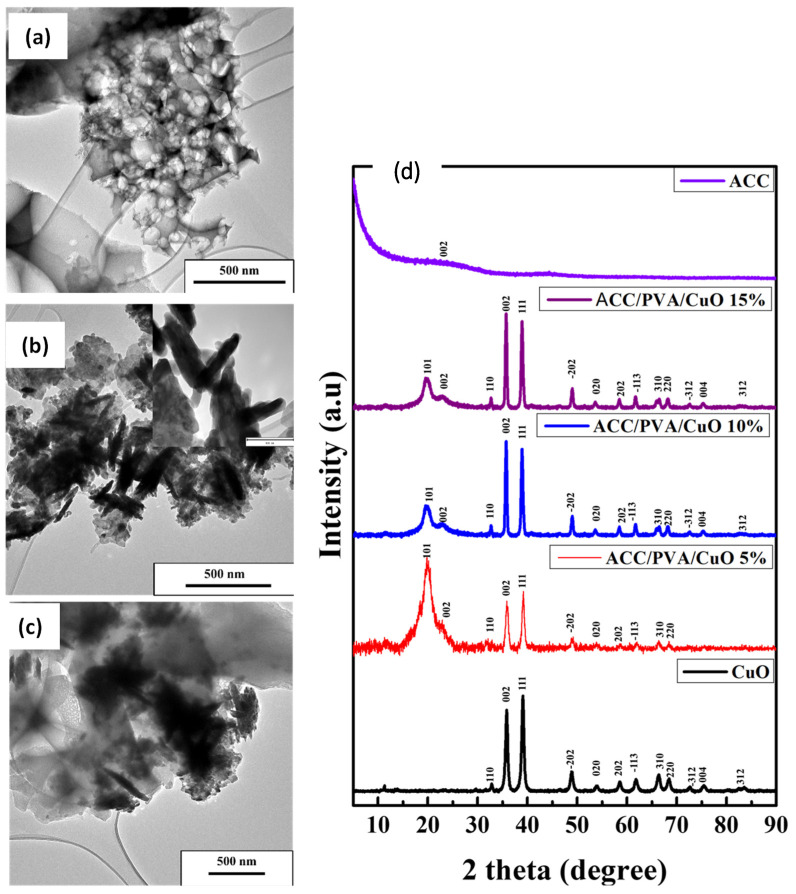
TEM images. (**a**) ACC, (**b**) CuO NPs, (**c**) ACC/PVA/CuO 5%, (**d**) XRD pattern of the prepared samples. The inset in (**b**) shows TEM images at a higher magnification scale.

To further investigate the as-prepared samples, the morphology of the materials was studied by TEM (Figure 1). The pristine activated carbon is shown in Figure 1a, exhibiting a porous structure. This can be attributed to the activation step and is a desirable property in sensing materials [10]. TEM analysis of CuO (Figure 1b) confirmed the presence of a flower-like sample comprised of agglomerated, poly-dispersed rods with diameters ranging from 8.31 to 52.41 nm. Similar properties were reported by Vasantharaj et al. [11]. The ACC/PVA/CuO composites show the deposition of the rod-like particles on the activated carbon matrix (Figure 1c). The crystallographic and structural characteristics of the synthesized CuO rods and ACC/PVA/CuO composites were evaluated using XRD, as shown in Figure 1d.

ACC exhibits broad and wide diffraction peaks at around 23.8° and 42.5°, confirming the (002) and (100) phase of the graphitic domain. These peaks demonstrate the formation of the small graphitic phase in AC during calcination. Comparison of XRD patterns of CuO and ACC/PVA/CuO samples in Figure 1d revealed that CuO exhibited a monoclinic structure that was indexed to CuO (PDF 01-073-6234) in all samples. All the observed peaks were assigned to the tenorite phase; in addition, a broad diffraction peak at 2θ range of 19.65° and a shoulder peak at 23.15° were observed in the case of ACC/PVA/CuO samples, confirming the presence of AC and PVA. The intensity of the CuO peaks in ACC/PVA/CuO tends to increase with increasing amounts of CuO as expected.

**Figure 2:**
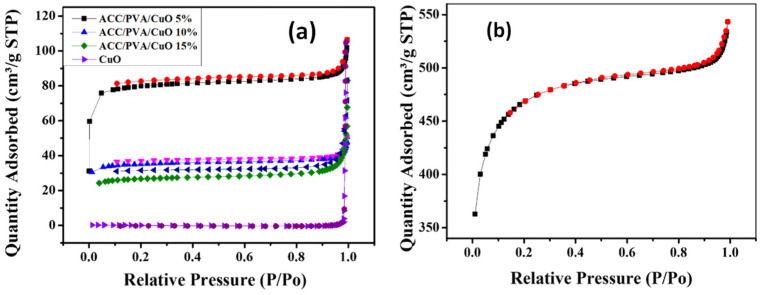
(**a**) N_2_ adsorption–desorption isotherms of CuO particles and ACC/PVA/CuO composites. (**b**) N_2_ adsorption–desorption isotherm of ACC.

Figure 2a,b shows the nitrogen adsorption isotherms measured for all samples. The ACC and the modified ACC/PVA/CuO composite samples exhibit type-IV isotherms according to the International Union of Pure and Applied Chemistry (IUPAC) classification. In each of the samples, the gas quantity adsorbed evidently increase at low relative pressure (P/P_0_ < 0.2) due to the abundance of meso-pores [12]. The ACC/PVA/CuO composites exhibited higher specific surface areas of 296, 132, 98 m^2^/g for ACC/PVA/CuO 5%, ACC/PVA/CuO 10%, and ACC/PVA/CuO 15%, respectively, when compared to the pristine CuO (0.62 m^2^/g). This increase in the surface area of the ACC/PVA/CuO composites can be attributed to the addition of ACC (1508 m^2^/g), which exhibits a much higher specific surface area. The nitrogen adsorption of the composites decreases gradually with an increase in the CuO loading, attributed to the fact that the pore channels of the ACC are occupied by the introduced CuO species, resulting in a reduction in the total pore volume and specific surface area compared to the pristine ACC sample [13]. The textural properties of the ACC, CuO, and ACC/PVA/CuO composites are summarized in Table 1.

**Figure 3:**
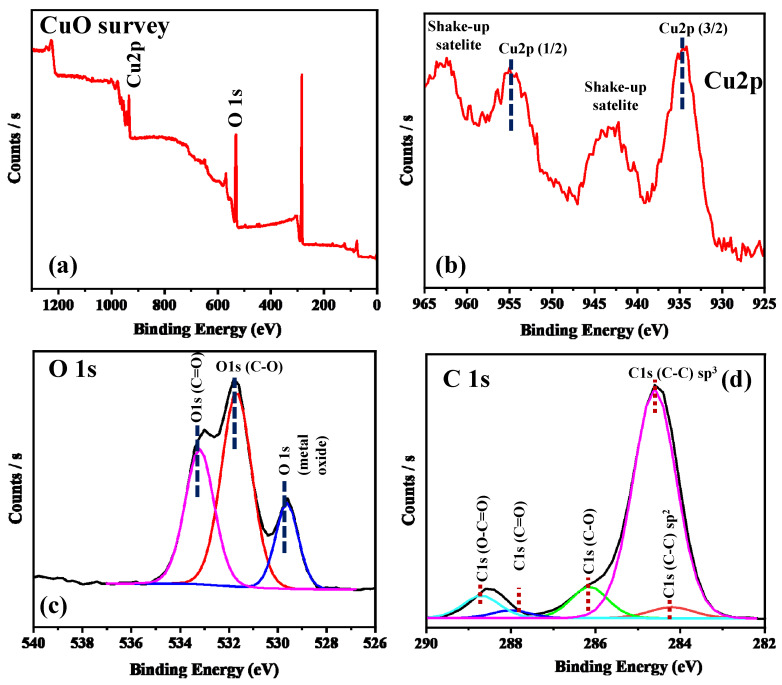
(**a**) XPS survey spectrum of CuO NPs. (**b**–**d**) High resolution (Core level) of Cu 2p, O 1s, and C 1s from as synthesized CuO NPs, respectively. The colored peaks in (**c**) and (**d**) show the deconvolution of the O1s and C1s peaks, respectively.

XPS analysis is a powerful surface sensitive technique that has been used to confirm the chemical composition, purity, and oxidation state of the as-prepared CuO NPS. Figure 3a shows the XPS survey spectrum of CuO NPs, which confirmed the presence of copper (Cu), oxygen (O), and carbon (C). Figure 3b–d shows the high-resolution spectra of Cu 2p, O1s, and C 1s, respectively. The high-resolution spectra of Cu 2p revealed distinguishable Cu 2p_3/2_ and Cu 2p_1/2_ peaks at binding energies of 934.6 and 954.6 eV, respectively, with an energy difference of 20 eV, which correlated well with the standard Cu 2p peaks for CuO [14]. In the same spectra, there is a satellite peak of Cu 2p_3/2_ at the higher binding energies allocated at 943.4 eV, and the presence of the Cu 2p satellite feature ruled out any possible Cu_2_O phase [15]. The high-resolution spectrum of O1s, as shown in Figure 3c, was deconvoluted to three peaks at 533.3, 531.7, and 529.6 eV. The peak at 529.6 eV, can be assigned to the binding energy for lattice oxygen (O_L_)^2−^ in the CuO lattice and is in good agreement with the binding energy of O^2−^ ion in the metal oxide sites (Cu^2+^−O^2−^) [16]. The peaks at 531.7 and 533.3 eV can be assigned to the binding energy for oxygen defects/vacancies (O_V_)^2−^ within the matrix of CuO, and the binding energy for adsorbed residual carbon or other surface oxygen species, which can easily react with the CuO NPS [17]. All the peaks of the C 1s spectrum, known as adventitious carbon contamination, are typically used as a charge reference for XPS spectra on the surface of the sample [16]. Figure 3d shows the high-resolution spectra of carbon (C 1s), which confirmed the presence of a reference peak at 284.7 eV and other peaks at 284.3, 286.1, 287.9, and 288.67 eV, assigned to the C–C sp^3^ bond, C=C sp^2^ bond, C=O, and O-C=O bond, respectively. The results measured from the XPS spectra confirmed the existence of the CuO structure.

For gas sensing investigations, the fabricated sensors were mounted in the test chamber for 30 min before exposure to analyte gases, in order to reach a stable electrical baseline [18]. Different VOCs (ethanol, acetone, 2-butanol, and methanol) were selected as test gases to demonstrate changes in selectivity and sensitivity of the ACC and the ACC/CuO composites, with exposure time of 5 min in vapor and 5 min in air. The gas response is presented as Response = ((R_a_ − R_g_)/R_g_) × 100%, where R_g_ and R_a_ are the resistances of the sensor under gas exposure and in air, respectively. All the gas sensors exhibited a higher response percentage to ethanol when compared to acetone, 2-butanol, and methanol, thus confirming their selectivity to ethanol. The major preference of activated carbon-based sensors for ethanol over the other is due to the presence of oxygen adsorption sites and dispersion interactions, mainly through the hydrocarbon chain on the activated carbon-based materials [19].

**Figure 4:**
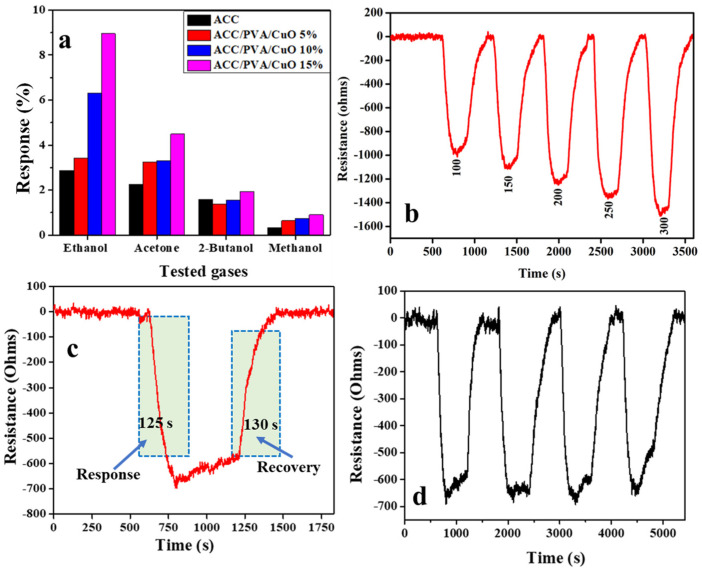
Sensitivity of the prepared sensors towards different VOCs at 300 ppm. (**a**); (**b**) Ethanol dynamic response curve for the ACC/PVA/CuO 15% sensor; (**c**) response and recovery time of ACC/PVA/CuO 15% to 100 ppm of ethanol gas; and (**d**) repeatability cycles of ACC/PVA/CuO 15% sensor towards 100 ppm ethanol at room temperature.

Figure 4 demonstrates that the sensor responses were dependent on the CuO concentration. The maximum sensor response for all the VOCs was achieved for ACC/PVA/CuO 15%, compared to pristine activated carbon and the other composites, in which ACC/PVA/CuO 15% > ACC/PVA/CuO 10% > ACC/PVA/CuO 5% > ACC. It is clear that the response of the composites is highly affected by the amount of CuO in the composite, as the response increased with CuO percentage loading. This confirms that the overall composite performance was improved by the addition of CuO, further confirming that the CuO nanoparticles can be effectively applied in room temperature as operable gas sensors when mixed with the ACC and PVA constituents.

Figure 4b illustrates the dependence of the sensor resistance on the ethanol concentration (ranging from 100–300 ppm) at ambient temperature for the ACC/PVA/CuO 15% sensor. The electrical resistance of the sensor decreased when in contact with ethanol and increased when exposed to air. Carbon materials and CuO are commonly p-type semiconductors and, for various sensor–analyte pairs, an inversion from p to n (or n to p) type response has been previously reported, where the n or p type here refers simply to the reaction to the analyte and not to the conductivity type (electrons or holes) [20]. Excellent reversibility with stable response and good recovery to the initial resistance state is observed, indicating good response and recovery processes, which are extremely important for a real-time sensing device. As ethanol concentration increases, ethanol could easily become adsorbed and react with the formed oxygen species on the surface of the sensor device, thus facilitating the sensing reactions, and significantly promoting the response. This result demonstrates that ACC/PVA/CuO-based composites can be considered as a low cost sensor in applications such as breath analysis to screen drunk drivers at ambient temperatures. Response and recovery are the central parameters that define the performance of a chemical sensor. Response time is defined as the amount of time point) in the presence of a target gas, and the recovery time is the time taken for recovery of 90% of the final equilibrium value. The response and recovery of the required for a sensor’s resistance change in order to reach 90% of the maximum response (saturation ACC/PVA/CuO 15% were 125 and 130 s, respectively, when exposed to 100 ppm of ethanol. To determine repeatability regarding the sensing material, the ACC/PVA/CuO 15% sensor was exposed to 100 ppm of ethanol. As seen in Figure 4d, the sensor demonstrated a reversible and stable sensor behavior after four cycles.

**Figure 5:**
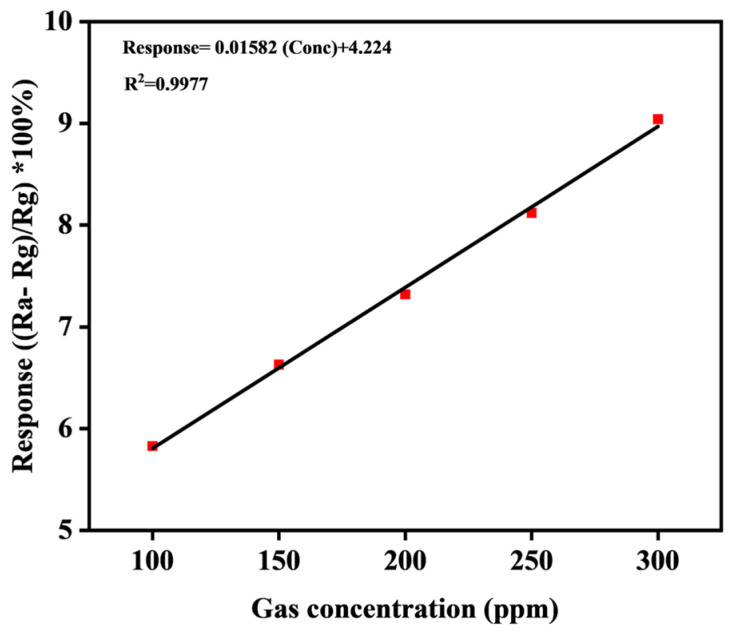
Linear relationship between response and gas concentration (100–300 ppm).

A liner fit of the sensor response vs. gas concentration is shown in Figure 5. The graph shows a linear relationship between the the response and gas concentration, with a high correlation when the test effluent increases from 100 ppm to 300 ppm, and with a correlation coefficient (R^2^) value of 0.9977. In this study, the limit of detection (LOD) for ethanol sensing was calculated at 10.27 ppm, using the formula LOD = 3σ/s (where σ is the standard deviation, and s is the slope of the linear response), which is comparable to previous studies, as shown in Table 2. The performance of the ethanol gas sensor in this work demonstrated significant improvement when compared to previous studies. Operating at room temperature (25 °C), the sensor achieved a lower LOD than that of graphene-based sensors (78.76 ppm) [21] and SnS/MoSe_2_ [22] heterojunction sensors (50 ppm) at the same temperature. The ZrO_2_/Co_3_O_4_ [23] sensor exhibited the lowest LOD (4.85 ppm); however, a much higher operating temperature of 200 °C was used. The α-Fe_2_O_3_ sensors [24], though widely studied, perform at an elevated temperature of 225 °C with a significantly higher LOD of 50 ppm. The ACC/PVA/CuO 15% possesses energy efficiency by eliminating the need for elevated temperatures, making it a promising candidate for ethanol gas detection applications.

With this limit of detection, the sensor has a potential to be used for breath alcohol detection, environmental monitoring, and quality control in industrial settings. For context, in breath alcohol analysis, a blood alcohol concentration (BAC) of 0.05–0.08%, which translates to 130–208 ppm in human breath, is the legal driving limit in many countries [25]. Thus, this sensor’s LOD is sensitive enough to detect ethanol concentrations significantly below the legal driving limit, making it suitable for early alcohol detection in law enforcement or personal breath analysers.

## 3. Materials and Methods

### 3.1. Synthesis of ACC

**Figure 6:**
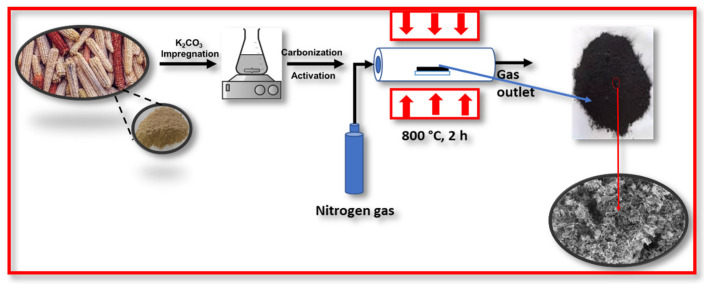
Schematic illustration of the steps taken to produce ACC from raw corncobs.

Initially, the raw corncob (CC) was washed with distilled water to remove impurities. It was then dried at 100 °C for 6 h before grinding into a fine powder using a kitchen blender. The obtained CC powder was directly mixed with a chemical activator (solid phase potassium carbonate) at an optimised weight ratio of CC/K_2_CO_3_ = 1:2. The mixture was placed on a quartz boat inside a tubular furnace that was heated at 800 °C (heating rate: 10 °C/min) under N_2_ atmosphere for 2 h. The furnace was then allowed to cool to room temperature and the resulting sample was washed several times with 1M HCl and distilled water. The sample was dried in an oven at 100 °C for 12 h. Figure 6 demonstrates the ACC synthesis graphically.

### 3.2. Synthesis of CuO

In a typical reaction, 2 M aqueous solution of NaOH was mixed slowly with 50 mL of 0.2 M aqueous solution of copper nitrate hexahydrate at room temperature and until a pH value of 12 was maintained. The mixture was then stirred for 12 h at room temperature. The resultant solution was sonicated for 30 min at room temperature, and the black coloured precipitate obtained was washed with distilled water and ethanol several times and dried at 100 °C for 6 h. Finally, the sample was calcined at 400 °C in a muffle furnace for 2 h.

### 3.3. Preparation of ACC/PVA/CuO

Briefly, 50 mg ACC was ultrasonicated in 10 mL dimethylformamide for about 15 min to allow particle dispersion. About 5 mg of commercial PVA was added to the mixture to form a continuous network and to bed the dispersed particles, and the resulting mixture was stirred at 60 °C for about 6 h to dissolve the PVA. The composite solutions were prepared in the following proportions: 5%, 10%, and 15% of CuO in ACC/PVA (*w*:*w*). For device fabrication, interdigitated electrode-printed circuit board (IDE-PCB) substrates were bought, with IDE already mounted on the PCB substrates. Two copper wires of the same size were mounted on each electrode by a soldering method. Prior to preparing the sensor films, the sensor substrate was rinsed with acetone and dried in a 100 °C oven for 2h. Preparation of sensor films was carried out by a drop casting method of the as-prepared composites using a micro-pipette. Precisely, 5 μL of the sonicated composites was carefully dropped on the sensing layer of IDE-PCB, and the sensors were allowed to dry at room temperature overnight and further dried in an oven at 60 °C for 4 h.

### 3.4. Gas Sensing Measurements

**Figure 7:**
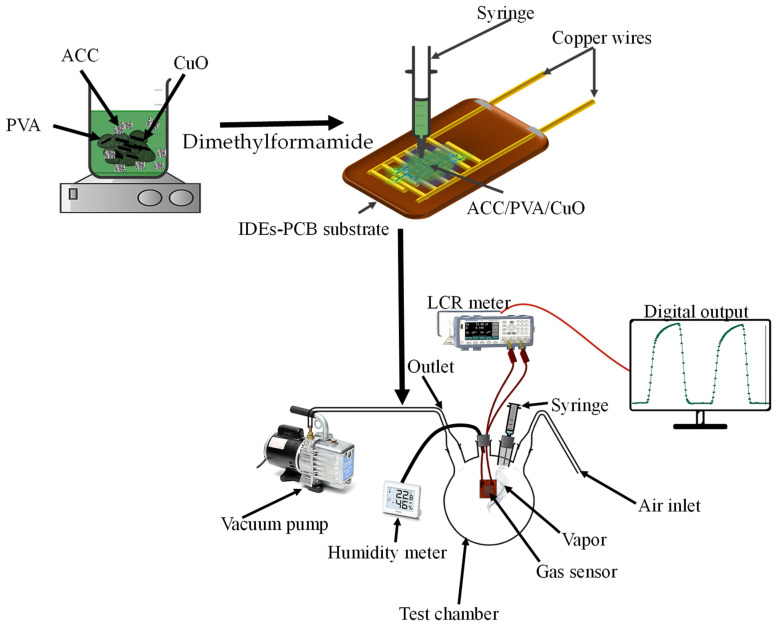
Graphical illustration of a home-made laboratory gas sensor set-up used to measure sensor response to VOCs.

The gas sensing properties of the fabricated sensing devices were studied by exposing the devices to the analytes (ethanol, acetone, 2-butanol, and methanol) and analysing the change in electrical resistance. An LCR 6300 multimeter with an AC input signal was used for the electrical resistance measurements. Figure 7 gives a schematic representation of the sensing system used, where the sensing electrode was placed inside a 5 L round bottom flask. The gas vapor of the different analytes was injected into the round bottom flask using a micro-syringe. To obtain the desired concentrations of analytes in ppm, the concentrations were calculated using Equation (1) [26]:

(1) $$(\mathrm{ppm})=\frac{22.4\;\rho TVs\;}{273\;MrV\;}\times 1000$$

where *C* is the concentration of the analyte gas in parts per million (ppm), *ρ* the density of the analyte in g∙mL^−1^, *T* the testing temperature in Kelvins, *Vs* the injected volume of the analyte in μL, *Mr* the molar mass of the analyte gas in g∙mol^−1^, and *V* the volume of the round bottom 5 L flask.

## 4. Conclusions

In this study, activated carbon (ACC) derived from corncob biomass waste, with a high specific surface area, was successfully prepared and applied in volatile organic compound (VOC) sensing at room temperature. The ACC/PVA/CuO composites with varying CuO concentrations demonstrated a preference for ethanol vapor, with the sensing response increasing along with increase in CuO content up to 15% CuO. The response and recovery times for the ACC/PVA/CuO 15% sensor in 100 ppm ethanol were 125 s and 130 s, respectively. Additionally, the sensor showed a low limit of detection (LOD) of approximately 10.27 ppm for ethanol, making it highly sensitive and suitable for applications such as breath alcohol detection, environmental monitoring, and quality control in industrial settings.

Future research should explore the effect of relative humidity on sensor performance, as well as the optimization of ACC/PVA/CuO composites for enhanced sensing capabilities. Furthermore, improving the long-term stability and reproducibility of the sensors by fine-tuning the synthesis process and exploring other composite materials could increase the overall performance for diverse VOC sensing applications, thereby expanding their utility in environmental and health-related fields. The design of an array of sensors that can distinguish between different analytes can help address the selectivity challenge, especially at low analyte concentrations.

## Figures and Tables

**Table 1:** Textural characteristics of activated carbon, CuO, and ACC/PVA/CuO composites.

	BET Surface Area (m^2^/g)	Pore Volume (cm^3^/g)	Pore Size (nm)
ACC	1508	0.72	2.17
CuO	0.62	0.12	1.44
ACC/PVA/CuO 5%	278	0.13	1.94
ACC/PVA/CuO 10%	133	0.061	1.83
ACC/PVA/CuO 15%	98	0.058	2.14

**Table 2:** Comparison of ethanol gas sensors based on temperature and LOD.

Gas Sensors Material	Operation Temperature (°C)	LOD (ppm)	Reference
PVA/ACC/CuO	25	10.27	This work
Graphene	25	78.76	[21]
SnS/MoSe_2_	25	50	[22]
ZrO/CoO_4_	200	4.85	[23]
α-FeO_3_	225	50	[24]

## Data Availability

Data is contained within the article.
